# Recent Advances of Salivary Gland Biopsy in Sjögren's Syndrome

**DOI:** 10.3389/fmed.2021.792593

**Published:** 2022-01-10

**Authors:** Rui Liao, Hai-Tao Yang, Heng Li, Li-Xiong Liu, Kai Li, Jing-Jing Li, Jie Liang, Xiao-Ping Hong, Yu-Lan Chen, Dong-Zhou Liu

**Affiliations:** ^1^The Second Clinical Medical College, Jinan University (Shenzhen People's Hospital), Shenzhen, China; ^2^Department of Rheumatology and Immunology, Shenzhen People's Hospital, The Second Clinical Medical College, Jinan University, The First Affiliated Hospital, Southern University of Science and Technology, Shenzhen, China

**Keywords:** Sjögren's syndrome, salivary gland biopsy, histopathology, mechanism, clinical significance

## Abstract

Sjögren's syndrome (SS) is a chronic, systemic, inflammatory autoimmune disease characterized by lymphocyte proliferation and progressive damage to exocrine glands. The diagnosis of SS is challenging due to its complicated clinical manifestations and non-specific signs. Salivary gland biopsy plays an important role in the diagnosis of SS, especially with anti-Sjögren's syndrome antigen A (SSA) and anti-SSB antibody negativity. Histopathology based on biopsy has clinical significance for disease stratification and prognosis evaluation, such as risk assessment for the development of non-Hodgkin's lymphoma. Furthermore, histopathological changes of salivary gland may be implicated in evaluating the efficacy of biological agents in SS. In this review, we summarize the histopathological features of salivary gland, the mechanism of histopathological changes and their clinical significance, as well as non-invasive imaging techniques of salivary glands as a potential alternative to salivary gland biopsy in SS.

## Introduction

Sjögren's syndrome (SS) is a chronic, systemic, inflammatory autoimmune disease characterized by lymphocyte proliferation and progressive damage to exocrine glands. In addition to impairment of salivary gland and lacrimal gland function, SS is frequently accompanied by multiple serum autoantibodies and systemic organ involvement that has a huge impact on the long-term quality of life of patients (1). The prevalence of SS in the general population is ~0.5% (2), with 0.33–0.77% reported in China (3).

Salivary gland histopathology based on salivary gland biopsy plays an important role in the diagnosis of SS and therefore broadly applied in clinical practice (4). Currently, minor salivary gland biopsy is the most common and the best method to determine the salivary gland composition in SS in clinical setting due to its disease specificity, wide availability, and minimal invasiveness. According to the classification criteria for SS, labial salivary gland biopsy (LSGB) is mandatory, especially with anti-SSA and anti-SSB antibody negativity (5–7). LSGB can also be used for disease stratification and prognostic evaluation. Therefore, in this review, we focus on the histopathological features and the mechanism of histopathological changes of LSGB, as well as their clinical significance in SS.

## Sampling Method of Salivary Gland Biopsy

The traditional biopsy method is primarily lip gland biopsy due to the size of the wound. The lower lip is easier to manipulate and the risk of excessive bleeding is very small, therefore, the lower lip is generally selected as the site for biopsy (8). The general recommendation is a 0.5–1 cm long fusiform incision from the lower lip mucosa to the muscle layer (9). The gland tissue should include at least four small salivary glands and the minimum gland surface area should be 8 mm^2^. If the salivary glands are too small (<2 mm), 6 glands should be taken (10).

By combining haematoxylin-eosin (H&E) staining with immunohistochemical staining, lymphocyte infiltration in LSGB can be well-identified, increasing the certainty of diagnosis. However, due to the high cost, immunohistochemistry is not widely used in clinical practice and may only be considered when the diagnosis is difficult or unclear (11). Sarioglu et al. considered that increasing the number of foci at multiple section levels and the total surface area to calculate the accumulative focus score might improve diagnostic performance and accuracy (10, 12). Detection of specific proteomic biomarkers in labial gland tissues can also help to improve diagnostic accuracy (13). Ultrahigh-frequency ultrasonography, a recently introduced diagnostic technique, may be implemented to guide LSGB by digital imaging and improve the detection rate (14).

## Histopathological Features of Salivary Glands

**Figure 1:**
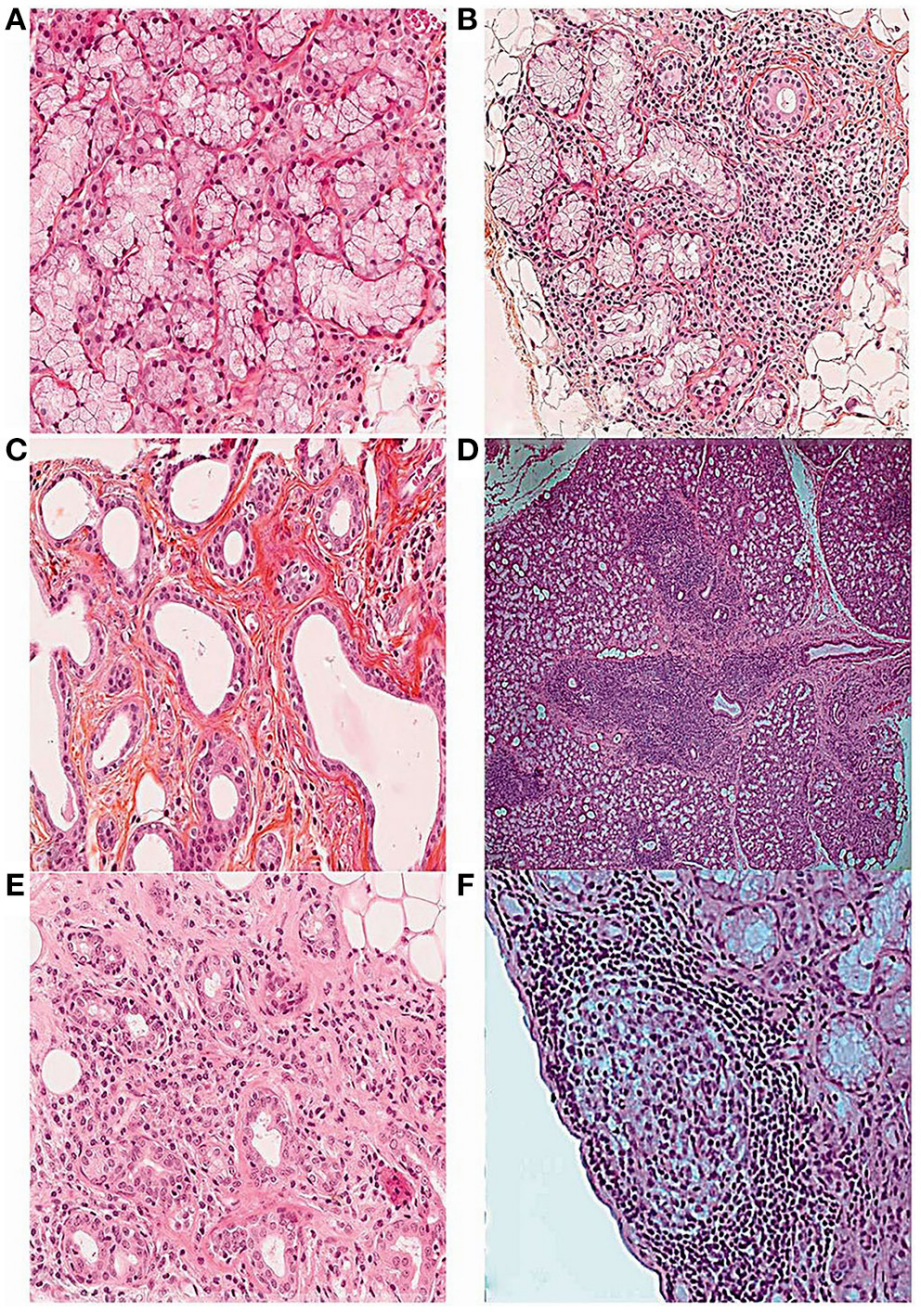
Histopathological features of the minor salivary gland biopsy in patients with SS stained with hematoxylin, eosin and saffron (16). **(A)** Normal salivary gland tissue (10×). **(B)** Focal lymphocytic sialadenitis (FLS) with perivascular or periductular aggregates of >50 lymphocytes (10×). **(C)** Dilated ducts of the minor salivary gland biopsy (10×). **(D)** FLS surrounded by normal gland tissue (4×). **(E)** Non-specific chronic sialadenitis (NSCS) with acinar atrophy, interstitial fibrosis and scattered mononuclear cell infiltrates (10×). **(F)** FLS with germinal center-like structure (10×).

The salivary gland is composed of parenchyma and stroma. The parenchyma contains a basic secretory unit, sebaceous gland and myoepithelial cells, and the basic secretory unit includes the acinus and duct system that consists of a moistening tube, secretory tube and excretory tube (15). Normal parenchyma with mucinous acini is shown in Figure 1A (16).

The histopathological features of salivary glands in SS include parenchymal and ductal changes. A decrease or even disappearance of acini, lymphocyte infiltration and proliferation of the lining cells, and formation of epimyoepithelial cell islands can be observed in the salivary glands of patients with SS (8). Furthermore, focal inflammation in salivary gland tissue is usually accompanied by acinar atrophy, ductal dilatation, and fibrosis. Another prominent feature is the presence of adipose tissue, the significance of which for the pathology of the labial gland is still controversial (17). Lobular fibrosis, often ignored in labial gland pathology, is also related to the severity of inflammation (18).

Although the mechanism triggering salivary gland inflammation remains unclear, some studies suggest that virus infection or tissue damage may lead to activation of the innate immune pathway and apoptosis of epithelial cells (19). During innate immune pathway activation, stimulated transcriptional profile of plasmacytoid dendritic cells (pDCs) underlies the development of sialadenitis, and type-I interferon (IFN) is considered to be the key mediator. pDCs are premier type-I IFN-producing cells that trigger production of type-I IFN and other proinflammatory cytokines after recognizing viral RNA and DNA, significantly increasing the level of type-I IFN and therefore amplifying inflammation. In addition, type-I IFN stimulates lymphocyte aggregation and proliferation by activating classical dendritic cells (DCs) and other immune cells (20). An increased infiltration of DCs and macrophages in local lesions will aggravate damage to salivary gland tissue as well as gland function disorder and is related to the occurrence of lymphoma (21).

Antigen-driven T cell-mediated B cell activation and hyperfunction are markers of SS (22). T cells are dominant in mild lesions, whereas B cells are the most representative subsets in advanced lesions (23). Among T cell subsets involved in the pathogenesis of SS, follicular helper T (Tfh) cells are key mediators of T cell-dependent B cell hyperactivity. Tfhs primarily promote the T cell-dependent B cell response in germinal center (GC)-like structures by secreting interleukin (IL)-21, driving B cell activation and differentiation into plasma cells. Tfhs can promote secretion of proinflammatory cytokines in target tissues, such as IL-17 and IFN, participating in the specific pathological functions of different stages of SS development (24, 25). Various studies have shown that CD8^+^ T lymphocytes also contribute to damage to exocrine acini (26). On the other hand, a large number of B cells proliferate and become activated in the salivary glands. Epithelial and immune cells in the salivary gland produce a large number of cytokines and chemokines, such as B-cell activation factor (BAFF), and thereby promote recruitment, activation, differentiation, and survival of B cells. Notably, imbalance and disorder of B cell subsets has an important role in driving inflammation. B cell subsets, such as CD27^+^ memory B cells, marginal zone B cells and plasma cells, increase and become further activated in salivary glands, though the function of regulatory B cells with inhibitory immune function is decreased, resulting in persistent local inflammation and leading to gland tissue damage (22, 27).

Salivary epithelial cells, including acinar cells, ductal cells, and salivary gland progenitor cells (28), are key in the development of SS and in regulating the activation and differentiation of infiltrating immune cells (19). The imbalance of the innate immune signaling pathway in salivary gland epithelial cells leads to the production of various proinflammatory cytokines by epithelial cells and promotes the aggregation and infiltration of T cells and B cells into salivary gland tissue, which leads to salivary gland dysfunction (19). Salivary gland progenitor cells can proliferate and differentiate into new salivary gland cells after the destruction of acinar and duct epithelial cells to maintain the stability of the salivary gland environment. Studies have shown that the aging of salivary gland progenitor cells in SS patients may represent one of the mechanisms underlying salivary gland dysfunction, which may be an early feature of SS (29, 30).

Taken together, diagnosing SS at the early stage is critical to improve the clinical outcomes of patients with SS. Some recent studies have shown that salivary gland histopathology may have a unique advantage in identifying the early stage of SS before glandular function is obviously damaged (31). However, histopathological characteristics involved in different stages of SS are not very clear yet. Glandular epithelial alternations, as the initiators of autoimmunity in SS, should be one of the focuses in future studies of SS.

## Morphologic Patterns of the Labial Salivary Gland for SJÖGREN'S Syndrome

Several morphological patterns of chronic inflammation are observed in LSGB of patients with SS, including focal lymphocytic sialadenitis (FLS), GCs, non-specific chronic sialadenitis (NSCS), sclerosing chronic sialadenitis (SCS), and lymphoepithelial lesions (LELs). FLS is a specific manifestation of SS (32).

### Focal Lymphocytic Sialadenitis

FLS is defined as the presence of more than 50 lymphocytes around the blood vessels or ducts of the salivary glands (Figures 1B,D) (16), which are usually surrounded by normal gland tissue. The collection of each lymphocyte aggregation is called a focus. The focus score is the number of foci per 4 mm^2^ of the gland, and the presence of FLS in an LSGB with a focus score ≥1 focus/4 mm^2^ is the most specific criterion for the diagnosis and classification of SS (4, 32).

Aggregation of lymphocytes and B cell hyperactivity are the characteristic pathological features of FLS (33). Lymphocytes are attracted to the salivary gland *via* various proinflammatory cytokines and cytokines. CXCL10 is a major mediator in the early formation of lymphocytic infiltration around the ductal gland and is highly expressed in the ductal epithelium. With disease development, the chemokines CCL19, CCL21, and CXCL13 are highly expressed in the salivary glands of patients with SS. CCL19 and CCL21 bind to the CCR7 receptor, which recruits naive and memory T cells, B cells, DCs, and other immune cells to the salivary glands. Other cell types, such as pDCs and natural killer cells, are also present in different numbers, but they account for <10% of the total number of cells. CXCL13 binding to CXCR5 plays an important role in the aggregation of B cells (23, 33–36). CXCL12 production by epithelial cells in the salivary gland of SS patients can attract plasma cells that exhibit a long-lived phenotype, and the local salivary gland microenvironment provides niche factors for plasma cells to survive (35). Destruction of the endothelial barrier function creates favorable conditions for the migration and aggregation of these immune cells (37).

The existence of FLS is one of the hallmarks of SS, and a focus score >1 provides a semiquantitative assessment of SS salivary composition. Findings of a focus score =1 may represent an early or mild form of salivary components of SS, which should be suspected to the diagnosis of SS (38). Compared to patients with a focus score of FLS <1 or NSCS/SCS, a focus score ≥1 is more strongly associated with the main phenotypic features of SS, including positive anti-SSA/SSB and rheumatoid factor, high ANA titers and immunoglobulin (Ig)G concentration, presence of an ocular staining score ≥3, and unstimulated whole salivary flow rates <0.1 ml/min (32). Patients with a higher focus score are more likely to exhibit double positivity for anti-SSA and anti-SSB antibodies (39), and a retrospective cohort study showed that a high focus score is an independent predictor of deterioration of exocrine gland function (40). Notably, it was proven that patients with a higher focus score are more likely to develop salivary gland swelling and lymphoma (39). A focus score of FLS ≥3 is considered to be a significant independent factor for the occurrence of non-Hodgkin's lymphoma (NHL), and the number of focus scores is useful for identifying patients with an increased risk of lymphoma (41).

Nevertheless, there is not always a direct correlation between the degree of lymphoid infiltration and exocrine dysfunction, and a focus score ≥1 is not always associated with symptoms of dry mouth or dry eyes (32). Of note, studies have shown that a focus score >1 may also occur in up to 15% of normal controls (42), and even though a focus score <1 does not mean a lack of a lesion in the LSGB. It is not clear whether the lesions are evenly distributed throughout the salivary gland, and there may be a risk of sampling error (10). There are differences in the focus score evaluation methods, and focus score results are often misestimated; if necessary, multiple biopsies should be performed to reduce sampling errors, and the use of standard evaluation methods is essential (16). Furthermore, a single-center experience showed that smoking and the use of antihistamines can reduce the occurrence of FLS in clinical practice (43). Therefore, a thorough medication and smoking history of patients must be determined before LSGB.

To sum up, although FLS is the feature of the salivary gland in SS, there was no definite correlation between the severity of FLS and the destruction of the salivary gland function. Therefore, FLS may be more valuable in the diagnosis of SS rather than stratification of the disease.

### Germinal Centers

The structure of GC involves well-defined infiltration of mononuclear inflammatory cells, dominated by B and T cells, follicular DC networks, and high endothelial venules. After H&E staining, dense dark areas, and bright areas can be seen in the salivary gland under light microscope (10). The histopathological features of GC are shown in Figure 1F (16).

The formation of GCs occurs due to the stimulation of mature B cells by antigens, which activate B cells in peripheral immune organs, with the help of T cells, to enter into a proliferative state. The levels of circulating cytokines (IL-17, IL-15) and chemokines [macrophage inflammatory protein (MIP)-1α, MIP-1β] are significantly increased in GC-positive SS patients, indicating enhanced migration of immune cells and capability of attracting B cells, T cells, macrophages and DCs for local recruitment in the salivary glands (44). At the same time, autoantigens may also help stimulate epithelial cells to further secrete cytokines, thus aggravating inflammation (19). Furthermore, CXCL13, a B-cell chemoattractant, causes aggregation of malignant B cells and promotes the formation of GCs and even lymphoma (45).

As GCs play a key role in driving activation of B cells and T cells, it is likely that high frequencies of GCs will aggravate the lymphocyte infiltration in SS patients, which may lead to increased destruction of glandular structures (46). Studies have shown that the presence of GCs in salivary glands correlates positively with B cell hyperfunction and focus score, as well as circulating autoantibodies and systemic manifestations (47). Furthermore, the existence of GCs leads to the continuous activation of B cells, which increases the risk of lymphoma. Hence, GC-like structures exhibit good reliability for predicting the occurrence of lymphoma (48). A multicenter study showed that the presence of GC-like structures is associated with a 7.8-fold increased risk of lymphoma occurrence (49).

However, there are some challenges in the detection of GCs. Sometimes GCs have not completely formed the typical light microscopic performance, even though it has produced the corresponding pathological function. Experts suggest additional staining with CD21 to improve the detection rate (10). Furthermore, Haacke et al. reported that GC formation is not a risk factor for NHL but a reflection of high disease activity, because their controlled trial showed no significant difference in GCs between SS patients with and without parotid mucosa-associated lymphoid tissue (MALT) (the most common NHL subtype in SS) (50).

Overall, evidence has shown that GCs may represent a high disease activity of SS and a potential risk for lymphoma, while the conclusions are not consistent across studies. Therefore, the significance of GCs in the development of SS and the potential risk for lymphoma needs to be thoroughly studied.

### Non-specific Chronic Sialadenitis

NSCS is characterized by acinar atrophy, interstitial fibrosis, and ductal dilatation, along with scattered or focal infiltration of lymphocytes, macrophages, and plasma cells, which are usually not adjacent to normal acini and located in the lobules of acini (Figures 1C,E) (16). SCS is considered to be a late stage of NSCS, and the main features of SCS are acinar atrophy and interstitial fibrosis. Due to the similar pathological features, NSCS and SCS are classified as one group (16). Notably, NSCS and SCS are not uncommon in the general population and tend to increase with age (10, 32).

Studies have shown that patients with NSCS/SCS display xerostomia and abnormal unstimulated and stimulated salivary flow (38, 51). However, the coexistence of NSCS and FLS is a very common phenomenon in SS, in which lymphocyte infiltration or lymphocyte aggregation in NSCS may be easily confused with FLS, resulting in the rate of FLS being misestimated. Therefore, it is necessary to quantitively describe the degree of lymphocyte infiltration, atrophy and fibrosis when evaluating the pathological features of salivary glands in SS. The lesion that is not adjacent to the normal parenchyma cannot be attributed to FLS. Neither NSCS nor SCS should be included in the FLS to prevent affecting the diagnosis and evaluation of the disease (10, 52).

### Lymphoepithelial Lesions

LELs are the characteristic manifestations of typical duct lesions in the salivary gland of SS patients that result from infiltration of lymphocytes into the hyperplasia of basal cells of the duct. LELs are surrounded by monocyte-like B cells, sometimes mixed with tumor plasma cells. The severity of LELs correlates positively with the number of B lymphocytes in the salivary gland, suggesting that B cells play an important role in the formation process of LELs (53, 54). LELs are more common in the parotid gland, which may be associated with asymmetric swelling of the parotid gland (55). The presence of LELs in parotid biopsy often indicates the risk for the occurrence of lymphoma, and it can even be considered as the initial stage of lymphoma in some cases. Therefore, LELs have a unique advantage for predicting lymphoma, but the diagnostic value of LELs remains to be studied (56, 57).

### Adipose Tissue

**Figure 2:**
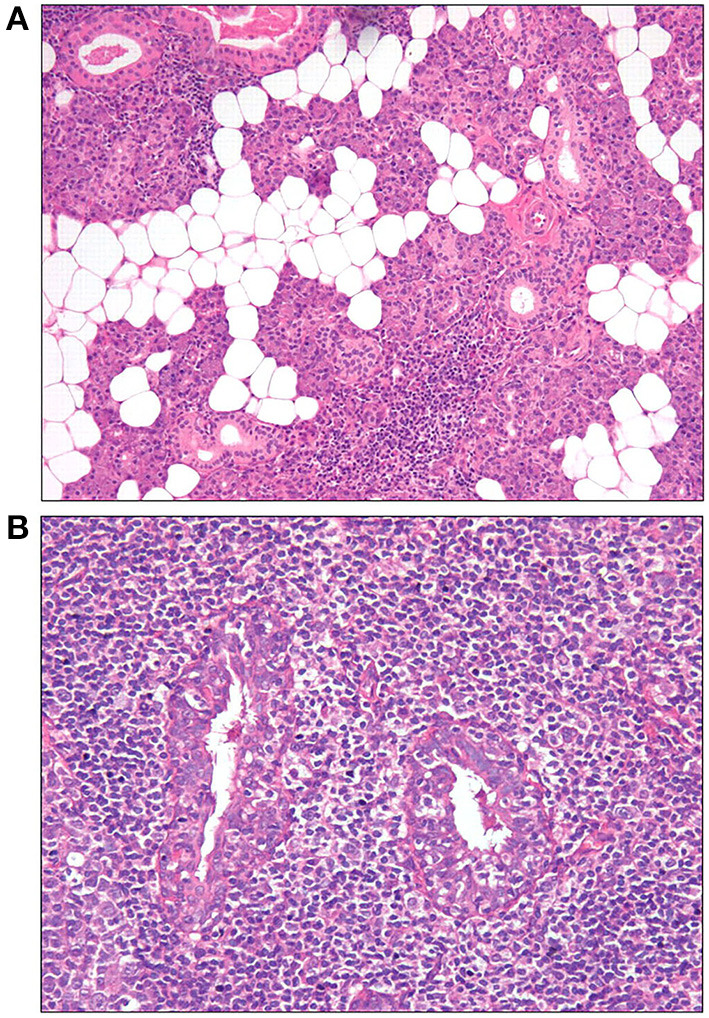
Histopathological features of the parotid gland tissue stained with hemoglobin and eosin staining (57). **(A)** Adipose infiltration and periductular lymphocytic infiltrates (10×). **(B)** Lymphoepithelial lesions (LELs) surrounded by lymphocytic infiltrates (20×).

Adipose tissue is defined as the replacement of normal glandular parenchyma by adipocytes (16, 57) (Figure 2A). The signaling involved in adipose tissue development in the salivary gland of SS is enhanced, and the numbers of autoantibody-specific B cells and plasma cells as well as the cytokine-rich regions are increased in the area of adipose tissue (58, 59). The increase in CD138^+^ plasma cells and CD20^+^ B cells correlates positively with fat infiltration and focal infiltration, indicating that adipocytes actively participate in promoting the inflammatory response (59). Patients with SS reportedly have more adipose tissue in their salivary glands than a healthy control group (17). The frequency of glandular dysfunction was significantly higher in those with adipose infiltration (60). However, one study has also shown that the composition of adipose tissue in labial gland increased significantly with age, indicating that it may be a feature of aging rather than a characteristic pathological manifestation of SS (61). Therefore, it remains unknown whether the development of adipose tissue is associated with the development of SS or merely represents a self-healing process in the body.

## Histopathology Grade

The first LSGB classification system, which was proposed in 1968, was divided into five grades according to the degree of lymphocyte infiltration (8). To quantify the number of labial gland lesions and to assess the relationship between the number of lesions and disease activity, Greenspan proposed the concept of FLS in 1974 (9), they suggest that the presence of FLS should be carefully assessed after the focus score has been calculated. In addition, the number of inflammatory cells, degree of acinar degeneration, fat infiltration, degree of fibrosis, the number of GCs and LELs were included in the evaluation. Moreover, the Sjögren's International Clinical Collaborative Alliance have then proposed a largely used protocol for sample preparation and focus score assessment in suspected SS^1^ (32).

Due to the diversity of the dynamic environment and interpretation of inflammatory glands, the pathological grading process for salivary glands is very complex. The main problems in the evaluation process include confusion between true FLS and non-lymphadenitis. FLS often coexists with NSCS as well as age-related chronic inflammatory responses and fat infiltration, presenting challenges for the identification and scoring of FLS (62). Some authors have proposed the total area and percentage of inflammatory infiltration as new histopathological parameters to improve the stratification of patients with SS. These two parameters can not only evaluate the infiltration degree of LSGB more accurately but also overcome the bias associated with focus score and ultimately improve the evaluation of SS (63). One obstacle of standardizing the assessment in focus scoring is error in calculating the salivary gland area of inflammatory infiltration by grid-based calculation. Lucchesi et al. proposed using digital image analysis to calculate the total area of the salivary gland, focus score, and inflammatory infiltration to reduce the error, which achieved far superior interrater agreement compared to a grid-based approach when calculating the total area (64).

## Clinical Significance of Labial Salivary Gland Biopsy in SJÖGREN'S Syndrome

A review conducted by Guellec et al. indicated that LSGB has a good diagnostic value for SS with an enhanced specificity and a sensitivity ranging from 63.5 to 93.7% (65). In healthy individuals, LSGB leads to a 6–9% false positive diagnosis. Moreover, 18–40% of patients with a clinical diagnosis of SS have a negative LSGB (66). Nevertheless, the frequency of abnormal LSGB improves with age. Post-mortem studies have shown that older age is associated with high false-positive rates of LSGB (67). Therefore, the specificity of LSGB in older patients is probably overestimated, and there is not always a definite correlation between lymphocyte infiltration and SS, especially in old patients. In contrast, a study that includes age as a control variable showed that the number of fibrotic tissues in the labial gland of patients with SS was significantly increased. The authors suggested that salivary gland fibrosis is an element of SS pathology related to FLS and is not solely attributable to age (18).

LSGB is recommended for a diagnosis of SS, especially in patients with anti-SSA and anti-SSB antibody negativity, which can be used for the early diagnosis of SS. The frequency of anti-SSA or anti-SSB antibodies is often lower in patients with SS who only have neurological involvement than in those without neurological involvement, and these patients often require salivary gland biopsy showing the presence of FLS for an early diagnosis of SS (4). IgA-expressing cells in the salivary gland are significantly decreased in SS, but IgG-expressing cells are significantly increased (68), and focus score combined with <70% IgA-expressing cells is considered to have higher sensitivity and specificity for the diagnosis of SS (69). Although LSGB is not the only gold standard for the diagnosis of SS, it is the best way to identify salivary components and evaluate the autoimmune activity. Routine LSGB may help to predict adverse outcomes, such as risk assessment for the development of NHL, and further assess the severity of the disease (32).

The severity and composition of lymphocyte infiltration in the salivary glands differ among individuals with SS. Intriguingly, it has been revealed that the pathological features of the salivary gland are fully developed at the time of diagnosis and remain stable thereafter. This indicates that the histopathological changes of the labial gland are quite stable over time, which may not reflect disease progression or disease activity (70). Some findings suggest that the major progression of salivary gland inflammatory lesions involves B cell malignant transformation rather than the type or number of infiltrating cells (71). In addition, fibrosis and fatty cell infiltration remain unchanged during a median time interval of 55 months, indicating that chronic inflammation in SS does not necessarily lead to degeneration of the glandular tissue or replacement with fibrosis and fatty cells (71). All these indicate that the histopathology of LSGB is not a perfect way to evaluate the progress or activity of the disease.

Until now, there is no specific immunosuppressive therapy for SS. Most international guidelines recommend symptomatic treatment for SS, such as local eye drops to relieve dry eyes or chewing to relieve symptoms of dry mouth. Immunosuppressants are used for systemic disease treatment intervention and management (72). Some believe that severe and long-standing disease of SS indicates irreversible dysfunction, thus treatment cannot effectively improve the symptoms of patients with dry mouth (51), and sometimes the effectiveness of treatment cannot be reflected by simple European League Against Rheumatism SS Patient Reported Index (ESSPRI) or European League Against Rheumatism SS Disease Activity Index (ESSDAI). Of note, pathological biopsy may reflect changes in glandular components during the process of disease development and treatment, representing a method to evaluate treatment efficacy (70).

According to the pathogenesis of SS, biological agents for the treatment of SS primarily affect the number of B cells, such as rituximab, or inhibit expression of BAFF, which is a target protein for B cell proliferation and/or activation, such as belimumab (19, 73). The primary mechanism of rituximab in the treatment of SS is depletion of B cells. Rituximab continuously reduced B cell infiltration and expression of IL-17 in the salivary gland and interfered with the formation of GC-like structures and LELs in the salivary gland. These findings show that SS patients have a good response to rituximab treatment (74–76). More importantly, some patients with SS and parotid gland-associated MALT lymphoma achieve complete remission after rituximab treatment, with plausible salivary gland tissue regeneration and functional recovery (77). Despite depletion of B cells in the salivary gland of patients treated with rituximab, Ig-producing cells in the salivary gland still exist (78), and continuous activation of B cells driven by BAFF may be the basis of relapse after treatment (79). Accordingly, a clinical trial found that FLS in the labial glands of some SS patients treated with belimumab for 28 weeks became negative; the B cell/T cell ratio and BAFF-positive cells in the labial salivary gland also decreased (80). However, another open-label study reported no significant change in the FLS of LSGB in SS patients treated with belimumab for 52 weeks (81). Although it has been proved that belimumab effectively reduces disease activity and peripheral serum biomarkers in SS (82), there is still insufficient evidence to support the FLS of LSGB as an indicator of the efficacy of belimumab treatment. Intriguingly, the combination of belimumab and rituximab for the treatment of SS can successfully achieve long-term clinical remission of lymphoma and cryoglobulinemia, indicating promising prospects for the alleviation of SS (83).

Janus kinase inhibitors constitute a potential novel therapy for SS that can reduce secretion of BAFF in the salivary gland and thereby decrease lymphocyte infiltration in the glands (84). Abatacept is a biological agent that inhibits T cell activation, reducing T cell-dependent B cell overactivity and CD4^+^ T cell subsets (85). Abatacept treatment could effectively improve xerostomia and reduced systemic disease activity in patients with SS (86). A preliminary study of abatacept for the treatment of SS showed that the inflammation of salivary glands and the number of lymphocytic foci were significantly reduced (87).

## Major Salivary Gland Biopsy

Major salivary gland biopsies, including parotid gland, sublingual gland, submandibular gland, and maxillary gland biopsy, have also been applied in the diagnosis of SS (88). Among these, parotid gland biopsy (PGB) is the most frequently reported. However, they are not commonly used in clinical practice, mainly due to risk of complications, such as facial nerve damage and development of salivary fistulae (66).

Notably, compared with LSGB, LELs are also characteristic histopathological changes in the major salivary glands and are more often observed in parotid gland tissue of patients with SS. The histopathological features of LELs in parotid gland tissue are shown in Figure 2B (57). When either a focus score ≥1 or a focus score <1 but with the presence of LELs is considered positive in parotid biopsy, the sensitivity and specificity of PGB and LSGB are comparable in the diagnosis of SS, at 78 and 86%, respectively (57). One early study by Marx et al. even showed that a significantly higher number of patients were confirmed by PGB than LSGB in patients suspicious for SS (100 vs. 58%) (89). In this study, lymphoma in five patients could only be identified by PGB but with no evidence by LSGB. Furthermore, Haacke et al. revealed that Fc receptor-like protein 4 (FcRL4), mainly expressed on some mucosa-associated B cells and MALT lymphoma B cells, correlated with the presence of LELs in the salivary gland of patients with primary SS. The level of FcRL4 mRNA in parotid MALT lymphoma was significantly higher than that in parotid gland from individuals without lymphoma. Moreover, FcRL4-positive B cells were far more common in PGB than those in LSGB, suggesting a potential role of FcRL4-positive B cells in the parotid gland as an indicator of SS-associated MALT lymphoma (54). In addition, PGB may have a better advantage in predicting early-stage lymphoma due to the fact that SS-associated lymphoma often primarily occurs in the parotid gland (50, 57, 90). Therefore, PGB is suggested to be an alternative to LSGB in SS.

The results of one randomized clinical trial have shown that rituximab treatment for SS leads to a major decrease in lymphocytic infiltration and the number of B cells, GCs and LELs in the parotid gland parenchyma (76). This study also revealed that the absolute pre-treatment number of CD20^+^ B cells/mm^2^ in the parotid gland parenchyma are predictive for the responsiveness to rituximab therapy in patients with SS as defined by ESSDAI, suggesting a potential role of PGB in guiding personalized treatment. Moreover, Haacke et al. demonstrated that the number of FcRL4-positive B cells is significantly decreased in the parotid gland after rituximab treatment, accompanied by restoration of the glandular epithelium (54), indicating the change of FcRL4-positive B cells in the parotid gland as a possible marker for the efficacy of rituximab treatment. Additionally, comparison between sequential PGBs from patients with primary SS pre- and post-rituximab treatment revealed that increased parotid gland flow and restoration of the salivary sodium content may be associated with the histopathological changes of reduced glandular inflammation and redifferentiation of lymphoepithelial duct damage (91). Furthermore, abatacept has also been shown to reduce the formation of GCs in the parotid gland (92). Dynamic histopathological changes in parotid gland may have advantages in determining response during treatment with biological agents due to the possibility of repeated biopsies in the same parotid gland (70). Therefore, PGB could also have a potential for diagnosis and evaluation of therapeutic response in SS.

## Salivary Gland Biopsy in Other Systemic Diseases

In addition to primary SS, histopathological changes in salivary glands are also observed in patients with other connective tissue diseases. Peculiar histopathological changes of LSGB in patients with systemic lupus erythematosus (SLE) may be a multisystemic presentation independent of SS (93). Lymphocyte infiltration mainly accumulates around blood vessels in LSGB of patients with SLE. Compared with those without perivascular infiltrates in LSGB, the presence of perivascular infiltration in patients with concurrent SLE and SS is significantly associated with longer durations of disease, sicca manifestations, and salivary gland enlargement (94). Notably, periductal infiltrates can be observed in the majority of patients with rheumatoid arthritis and sicca syndrome, while the frequencies of DCs and macrophages are increased in those patients with a focus score <1 and negative anti-SSA or anti-SSB antibodies (95), which is different from that of primary SS. Furthermore, sicca syndrome in systemic sclerosis (SSc) has been considered to be attributed to SSc-associated glandular fibrosis in LSGB, which is independent from SS-associated lymphocytic sialadenitis (96). However, further studies are still needed to evaluate the relationship between the histopathological changes of salivary glands and disease activity, as well as prognosis in different connective tissue diseases.

Other systemic diseases, such as IgG4-related diseases, sarcoidosis, and amyloidosis, have different characteristic histopathological features (97). The three main histopathological features of IgG4-related diseases are dense lymphoplasmacytic infiltrate, fibrosis (arranged at least focally in a storiform pattern) and occlusive phlebitis. The infiltrating lymphocytes are mainly composed of T cells and aggregates of B cells, with an IgG4^+^/IgG^+^ plasma cell ratio of >40% as a mandatory for histological diagnosis of IgG4-related diseases (98). Nevertheless, the main histopathological feature of sarcoidosis is non-caseous epithelioid-cell granulomatous lesions without the evidence of organisms or particles (99), and the presence of amyloid protein in the salivary gland can be of great diagnostic value in amyloidosis. Of note, Marx et al. demonstrated that PGB seems to have a higher diagnostic accuracy for sarcoidosis (93 vs. 36%) as well as sialosis with enlarged parotids (14 vs. 0%) compared to LSGB (89). One case report about a woman with concurrent hemochromatosis and sicca symptom revealed significant deposition of iron in acinar and duct epithelial cells without focal lymphocytic infiltration in the LSGB, and her iron deposition completely disappeared after desferrioxamine treatment (100). In addition, the affected salivary gland biopsy, mainly parotid and submandibular glands, has also been suggested in patients suspected with anti-neutrophil cytoplasmic autoantibody (ANCA)-associated vasculitis. The histological features, such as vasculitis, granulomatous inflammation, necrosis, and the existence of multinucleated giant cells could be found in salivary gland lesions caused by ANCA-associated vasculitis (101). Intriguingly, postmortem biopsies of salivary glands were positive for SARS-CoV-2 by immunohistochemistry, which were proven to be a target for SARS-CoV-2, with cytoplasmic and nuclear vacuolization as well as nuclear pleomorphism in the ductal epithelium and degenerative changes of the zymogen granules and enlarged nuclei in acinar cells (102). These indicate that saliva detection may be used as a diagnostic method for SARS-CoV-2. Therefore, the salivary gland biopsy also contributes to the differential diagnosis of these systemic diseases.

## Imaging Techniques of Salivary Gland

Imaging techniques, such as salivary gland ultrasonography (SGUS), sialography, magnetic resonance imaging (MRI), and positron emission tomography/computed tomography (PET/CT), have also been shown to contribute to the diagnosis of SS, evaluation of disease activity and prognosis in SS (103). As an invasive tool with risk of radiation exposure, sialography is not frequently used in clinical practice. Moreover, despite the potential applicability in SS-associated lymphomas, MRI and PET/CT are neither often used in SS due to a high cost and unproven correlation with histopathological features. Therefore, SGUS, with a high spatial resolution in superficial organs, has gradually emerged as a promising imaging technique, as it is non-invasive, economic, and more easily accessible.

SGUS is mainly used for examination of major salivary glands, such as parotid glands and submandibular glands. SGUS has been proven to be effective in the detection of typical structural changes in SS, with uneven hypoechogenic areas, hyperechogenic reflection and unclear salivary gland boundaries as the typical ultrasonic manifestations in SS (103). There are various available SGUS scoring systems, while no specific scoring system to date has been recommended as optimal. Although new definitions for developing a novel semiquantitative SGUS scoring system in SS have been recently developed with good inter-reader and excellent intrareader reliabilities (104), further studies are required to achieve evidence-based consensus.

Consistency between SGUS and LSGB or PGB has been investigated in a large number of previous studies. Normal-appearing SGUS was statistically associated with negative LSGB in patients with SS (105). SGUS scores were positively correlated with the focus score of LSGB (Spearman r = 0.61) (106), and abnormal SGUS (score 2 or 3) was associated with GC-like structures in LSGB that indicate a possibly higher risk for lymphoma development (107). A high diagnostic accuracy of SGUS was comparable to LSGB in patients with SS (108). Of note, in a cohort of 103 consecutive patients clinically suspected for SS, a slightly higher absolute agreement was observed between SGUS and PGB compared with LSGB (83 vs. 79%) (90). The accuracy of SGUS to predict PGB and LSGB outcome was good, with an area under the curve of 0.849 and 0.824, respectively. Additionally, real-time sonoelastography based on SGUS may be useful in identifying SS patients with fibrotic changes in salivary gland (109). Intriguingly, studies have revealed an add-on value of SGUS in improving the performance and feasibility of the 2016 classification criteria for SS (110). Some researchers suggested that LSGB is even not recommended for the patients with negative SGUS and anti-extractable nuclear antigen antibodies unless there are otherwise strong indications for SS (111). Furthermore, patients with pathologic SGUS have more systemic complications and higher disease activity, as well as a higher risk for lymphoma development (107). Notably, ultrasound-guided core needle biopsy has recently been considered to be a safe and useful procedure in SS patients with suspected salivary gland lymphoma (112). Therefore, SGUS has great potential for the management of SS and is therefore considered as a promising alternative to salivary gland biopsy.

However, due to the different SGUS scoring systems applied, the sensitivity and specificity of SGUS vary largely among different studies. A systematic review has revealed a sensitivity of 45.8–91.6% and a specificity of 73–98.1% for SGUS in patients with suspected SS (113). Moreover, Mossel et al. found it only fair to moderate the correlation between SGUS score and focus score in parotid biopsy (Spearman r = 0.376) and LSGB (Spearman r = 0.412), respectively (114). The histopathological features of salivary glands, such as inflammatory infiltrates, might not be well-represented by the changes of hypoechogenic areas on SGUS. Whether or not the SGUS scoring system is sensitive enough to identify the changes is still unclear. Therefore, further studies are required to standardize the SGUS definition and to confirm the reliability of SGUS in SS, especially in identifying the early stage of SS (113). Long-term longitudinal studies, with clinical and histological records as well as imaging, are pivotal to clarify the correlation of SGUS and salivary gland biopsy.

## Conclusions

Histopathology of salivary glands, especially LSGB, not only plays an important role in the diagnosis of SS but also has a role in the stratification and prognosis of the disease. Moreover, it may have a unique advantage in identifying the early stage of SS. The degree of lymphocyte infiltration and focus score in pathological tissues often correlate positively with the disease activity of SS, while whether it represents a reliable indicator for the prognosis of SS is still controversial. In addition, GCs and LELs in salivary gland pathology may have potential significance in predicting lymphoma. Despite various clinical trials showing that biological agents cannot improve the symptoms of patients, the pathological changes of salivary glands indicate that biological agents do in fact ameliorate the damage and inflammatory infiltration of salivary glands, which suggests that salivary gland pathological changes may be useful for evaluating the efficacy of therapies in clinical trials.

Although LSGB is widely used in clinical practice, as an invasive method, the limitations of LSGB, including sampling errors and subjective errors in assessing histopathological patterns, cannot be completely neglected. Therefore, the acquisition of salivary gland tissue and histopathological interpretation as well as histopathological grading systems are required to be standardized for use in clinical practice. Moreover, non-invasive imaging techniques, especially SGUS, could be considered as a potential alternative to salivary gland biopsy in SS. Incorporation of SGUS into the classification criteria for SS may also have an add-on value in the diagnosis of SS.

## Author Contributions

RL and H-TY mainly drafted the manuscript. D-ZL and Y-LC conceived the work and revised the manuscript. HL, L-XL, KL, and J-JL participated in drafting the manuscript. JL and X-PH participated in editing the manuscript. All the authors contributed to the final manuscript.

## Funding

This work was supported by the National Natural Science Foundation of China (Grant Number: 81971464) and the National Key Research and Development Program of China (Grant Number: 2019YFC0840603).

## Conflict of Interest

The authors declare that the research was conducted in the absence of any commercial or financial relationships that could be construed as a potential conflict of interest.

## Publisher's Note

All claims expressed in this article are solely those of the authors and do not necessarily represent those of their affiliated organizations, or those of the publisher, the editors and the reviewers. Any product that may be evaluated in this article, or claim that may be made by its manufacturer, is not guaranteed or endorsed by the publisher.

## Footnotes

^1^ http://sicca.ucsf.edu/Labial_Salivary_Gland_Assessment.doc
